# Aggregation of Omic Data and Secretome Prediction Enable the Discovery of Candidate Plasma Biomarkers for Beef Tenderness

**DOI:** 10.3390/ijms21020664

**Published:** 2020-01-19

**Authors:** Sabrina Boudon, Joelle Henry-Berger, Isabelle Cassar-Malek

**Affiliations:** 1 Université Clermont Auvergne, INRAE, VetAgro Sup, UMR Herbivores, F-63122 Saint-Genes-Champanelle, France; sabrina.boudon@inra.fr; 2 Université Clermont Auvergne, GReD, UMR CNRS 6293–Inserm U1103, 63001 Clermont-Ferrand, France; joelle.henry@uca.fr

**Keywords:** data aggregation, secretome, plasma proteome, biomarkers, meat tenderness

## Abstract

Beef quality is a complex phenotype that can be evaluated only after animal slaughtering. Previous research has investigated the potential of genetic markers or muscle-derived proteins to assess beef tenderness. Thus, the use of low-invasive biomarkers in living animals is an issue for the beef sector. We hypothesized that publicly available data may help us discovering candidate plasma biomarkers. Thanks to a review of the literature, we built a corpus of articles on beef tenderness. Following data collection, aggregation, and computational reconstruction of the muscle secretome, the putative plasma proteins were searched by comparison with a bovine plasma proteome atlas and submitted to mining of biological information. Of the 44 publications included in the study, 469 unique gene names were extracted for aggregation. Seventy-one proteins putatively released in the plasma were revealed. Among them 13 proteins were predicted to be secreted in plasma, 44 proteins as hypothetically secreted in plasma, and 14 additional candidate proteins were detected thanks to network analysis. Among these 71 proteins, 24 were included in tenderness quantitative trait loci. The in-silico workflow enabled the discovery of candidate plasma biomarkers for beef tenderness from reconstruction of the secretome, to be examined in the cattle plasma proteome.

## 1. Introduction

Animal products are the main source of protein and essential nutrients in human nutrition. While in developing countries, the objective is to increase meat production to meet human nutritional needs, in industrialised countries the major expectations concern meat quality [1] A challenge for the beef sector in those countries is to predict and manage the meat quality attributes in order to ensure their low variability. Among the attributes of beef eating quality (tenderness, juiciness, flavour and colour), tenderness is a top priority for the beef industry to meet consumers’ expectations [2] However, beef tenderness is a complex phenotype with large individual variation within and between animals that can vary according to multi-factorial influences. Factors related to the animal itself including genotype [3] and physiological type (breed, age, and sex) [4,5,6] contribute to the variability in tenderness. Extrinsic factors include management systems and rearing conditions [7,8,9], animal transport and handling during the pre-slaughtering period, slaughtering conditions [10], and post-slaughter factors including maturation, storage and cooking [4,11].

Today, meat tenderness attributes are assessed only after animal slaughtering and meat ageing which limits the delivery of consistent quality meat [12,13,14]. Thus, the identification of biomarkers for meat quality measurable in living animals is a good opportunity to develop monitoring, decision-making and management tools for beef quality prior to slaughter. Thanks to genomics, several research groups have investigated the potential of muscle-derived markers for characterizing the molecular mechanisms underlying beef tenderness as well as for prediction purpose. Some DNA polymorphisms and transcript abundances were related to variation in tenderness. Thus, markers linked to genetic polymorphism were identified in proteolytic genes e.g., *CAPN1, CAST* [15,16] and marketed as genetic tests. Transcriptional muscle profiling enabled the detection of gene transcripts involved in fat, energy metabolism and heat shock response (e.g., *DNAJA1, HSPB1* and *CRYAB*), as candidate biomarkers for meat tenderness [17,18], which were included in a dedicated micro-array [18]. The development of proteomics has taken the issue of identification of tenderness biomarkers a step further [19,20]. Proteomic studies confirmed the importance for meat tenderness of proteins involved in muscle structure, energy metabolism, proteolysis or apoptosis (for a review, Picard et al. [21]). However, a high variability in muscle biomarker content is detected among breeds, individuals and muscles [22]. In addition, inverse relationships between some biomarkers and beef tenderness were also reported as a function of muscle properties [23].

So far, biomarker assessment requires muscle sampling in slaughtered animals or biopsies on living animals. Thus, the identification of generic and low invasive biomarkers in body fluids is an issue for molecular phenotyping in living animals [24]. As circulating proteins mirror the individual’s physiology, identification of plasma biomarkers could allow prediction of the tenderness potential of living animals. In this study, we hypothesized that the aggregation of public data may help to identify candidate plasma biomarkers for beef tenderness from the secretome of muscle. We thus designed a workflow to generate a dataset of known biomarkers for tenderness and predict in silico the proteins secreted through conventional pathways or other pathways allowing transit of proteins from muscle to the plasma.

## 2. Results

### 2.1. Literature Search and Data Aggregation

**Figure 1:**
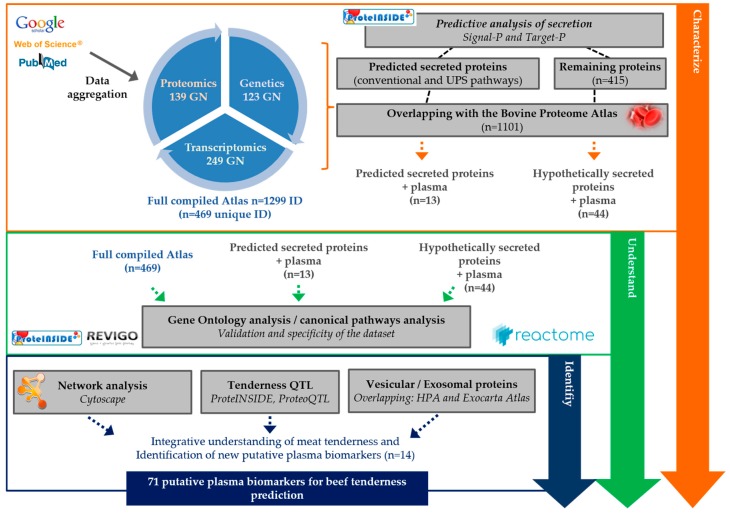
Flowchart of the workflow applied for the discovery of candidate plasma biomarkers for beef tenderness using a review of the literature and aggregation of omic data

**Figure 2:**
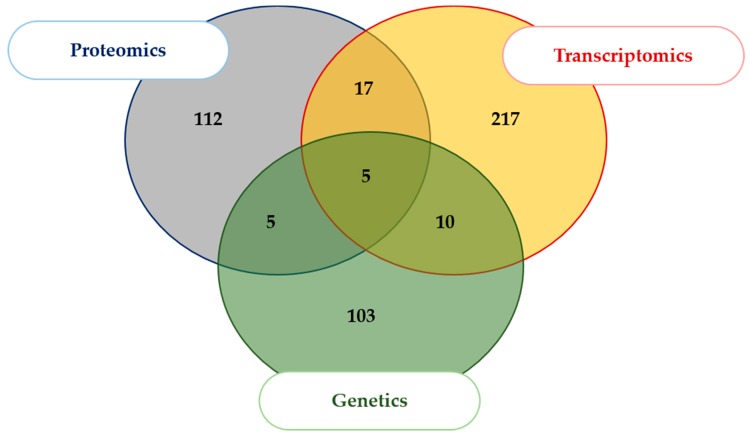
Origin of the omics data included in the study. The Venn diagram shows the intersects of the three omic datasets aggregated in the study. The aggregated dataset related to tenderness [64] was limited to the unique ID Gene Names.

A total of 459 articles including one GSE were identified using the MEDLINE, GOOGLE and CLAVIRATE analytics as related to meat tenderness (Figure 1). Among them, 425 articles were excluded because they did not meet the criteria of inclusion. From the corpus of the 44 remaining publications, 26 articles were identified as eligible for proteomic data [17,23,24,25,26,27,28,29,30,31,32,33,34,35,36,37,38,39,40,41,42,43,44,45,46,47]. Eleven articles including the series accession number GSE9256 (PMID: 18443416) were found as eligible for transcriptomic data [15,17,18,24,41,48,49,50,51,52,53]. Twelve articles were found as eligible for genetic data [18,50,54,55,56,57,58,59,60,61,62,63]. The computational data aggregation from these 44 publications gave an overview of 1299 ID gene name (GN) related to meat tenderness whatever the muscle, breed, animal type, sex, age at slaughter, geographic area, and methodologies used for tenderness evaluation. Depending on the type of molecule studied (protein, transcript or gene): 139 unique GN were reported as proteomic data, 249 unique GN as transcriptomic data, and 123 unique GN as genetic data. The compilation of these three lists generated the aggregated dataset comprising 469 non-redundant GN (Table 1, Figure 2).

### 2.2. Computational Prediction

Prediction of the secreted proteins. Table 1 illustrates the numbers and characteristics of the proteins associated with the omics datasets. The predictive analysis using ProteINSIDE from the aggregated dataset allowed us to identify 54 proteins (11.5%) as predicted secreted proteins according to a conventional pathway (with signal-P and/or TM domain) and 36 proteins (7.7%) as predicted secreted proteins according to UPS pathways (without signal-P). The list of remaining proteins included 379 GN (80.8%). 

Prediction of the secreted proteins putatively found in the plasma. The intersection of the datasets and the Bovine Plasma proteome Atlas (BPA) allowed to retrieve proteins that may be secreted by conventional or by UPS pathways and found in the plasma, and the remaining proteins not hallmarked for secretion but found in the plasma respectively (Table 1). Thirteen proteins referred to as “predicted secreted proteins in plasma” (2.8%) and 44 proteins referred to as “hypothetically secreted proteins in plasma” (9.4%) were identified respectively (Table 1). These repertoires are presented in Table 2.

### 2.3. Gene Ontology

**Figure 3:**
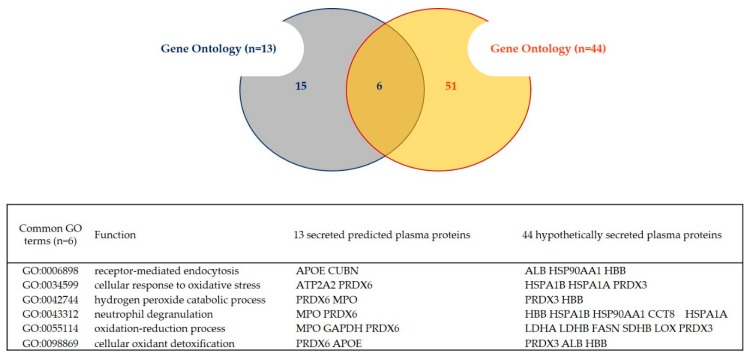
Comparison of the list of Gene Ontology terms identified in the 13 secreted plasma proteins + plasma and 44 hypothetically secreted proteins + plasma.

The full compiled atlas of 469 GN and the repertoires of 13 “predicted secreted proteins in plasma” and of 44 “hypothetically secreted proteins in plasma” were then submitted to Gene ontology (GO) annotation. The biological processes (BP) associated with the different datasets are presented in the Table 3, Table 4 and Table 5 respectively. The hierarchical “varonoi” visualization of the canonical pathways related to the 13 “predicted secreted proteins in plasma” and the 44 “hypothetically secreted proteins in plasma” are shown in Supplementary Data 1 and 2. A SimRel semantic rapprochement performed on the TOP50 of the GO terms associated with the 469 proteins (*p*-value adjusted <0.001, minimum of two proteins annotated in annotation) highlighted 10 BP: “Inflammatory response”, “Gluconeogenesis”, “ Protein stabilization”, “chaperone-mediated protein complex assembly”, “Carbohydrate metabolism”, “Aging”, “Muscle contraction and development”, “cell adhesion”, “protein folding” and “Apoptotic process” (Table 3). Thanks to REVIGO semantic rapprochement performed on the GO terms associated with the 13 “predicted secreted proteins in “plasma” (*p*-value adj. <0.05, minimum of two proteins annotated in GO annotation), s BP were identified: “Cell adhesion”, “Apoptotic process”, “Endocytosis”, “Response to oxidative stress”, “Hydrogen peroxide metabolism” and “Lipid metabolism” (Table 4). In parallel, thanks to the Reactome visualization of the 13 “predicted secreted proteins”; four major canonical pathways were identified: “homeostasis”, “signal transduction (receptor tyrosine kinase signaling, and NR1H2/H3 mediated signaling)”, “immune system (neutrophil degranulation)” and “transport of small molecules (plasma lipoprotein assembly, remodeling, ABC transporter ion channel, mitochondrial calcium ion transport) (Supplementary Data 1). Thanks to semantic rapprochement performed on the GO terms associated with the 44 “hypothetically secreted proteins in plasma” (*p*-value adj. <0.001, minimum of two proteins annotated in GO annotation), 9 BP were identified: “Protein stabilization”, “Gluconeogenesis”, “response to ethanol”, “Protein folding and chaperone-mediated protein complex assembly”, “Endocytosis”, “Muscle contraction”, “Viral process” and “Hydrogen peroxide metabolism” (Table 5). In parallel, thanks to the Reactome visualization of the 44 “hypothetically secreted proteins in plasma”; 10 major canonical pathways were identified: “cell-cell communication”, “homeostasis”, “muscle contraction”, “metabolism of proteins”, “metabolism of lipids (citric acid cycle and carbohydrate metabolism)”, “programmed cell death”, “cellular responses to external stimuli”, “organelle biogenesis and maintenance (cilium assembly…)”, “autophagy”, “extracellular matrix organization” (Supplementary Data 2). The comparison between the repertoires of 13 “predicted secreted proteins in plasma” and of 44 “hypothetically secreted proteins in plasma” revealed six common GO Biological Process including “receptor-mediated endocytosis”, “cellular response to oxidative stress”, “hydrogen peroxide catabolic process”, “neutrophil degranulation”, “oxidation-reduction process” and “cellular oxidant detoxification” (Figure 3).

### 2.4. Network Analysis and Plasma PPi Identification

**Figure 4:**
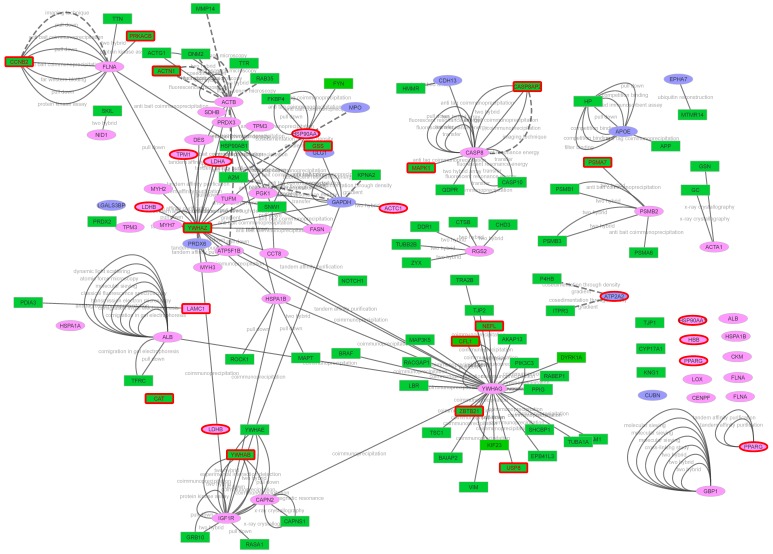
Network of the 71 plasma proteins identified in this study as putative candidate biomarkers for beef tenderness. This network reports the 71 plasma proteins identified as candidate biomarkers for meat tenderness in this study. The 13 predicted secreted proteins in plasma (conventional and alternative pathways) are shown in purple ellipse. The 44 hypothetically secreted proteins in plasma are shown in pink ellipse. The green rectangle refer to interactor identified through the up to date Cytoscape tool (MINT resource, Psciquic web service, 2017-12-17). The border red rectangle refer to the interactors located in cattle meat QTL for Shear force and/or Tenderness score tenderness (ProteINSIDE ProteoQTL analysis). Solid line shows the “primary interaction type”. Dotted line shows the interaction through “detection method”. Eleven out of the 57 plasma candidates (ATP5B, BPGM, COL11A1, COL13A1, ENO3, FGF12, LRRC16A, PCDH7, PGAM2, PVALB and TG), not included in the MINT database, are not shown in this network.

Examination of the network built from all of the 57 plasma candidates identified in this study (13 “predicted secreted proteins in plasma” and 44 “hypothetically secreted proteins in plasma” combined) revealed 544 interactors of which 75 proteins were present in the BPA (Figure 4). Eleven proteins out of the 57 plasma candidates (ATP5B, BPGM, COL11A1, COL13A1, ENO3, FGF12, LRRC16A, PCDH7, PGAM2, PVALB and TG) were not included in the MINT database used to generate the network from Cytoscape. Finally, the investigation of these 75 candidate proteins allowed to identify 14 additional proteins (CASP8AP2, ZBTB21, USP8, NEFL, CAT, GSS, PRKACB, CFL1, MAPK1, CCNB2, ACTN1, YWHAZ, YWHAB and PSMA7) that could be new meat tenderness proteins located in cattle meat Quantitative trait *loci* (QTL) for Shear force and/or Tenderness score (Table 2). These 14 proteins were included in the repertoire of the “secreted proteins in plasma”. Thus, a repertoire of 71 non-redundant candidate plasma proteins related to tenderness was generated (Table 2).

### 2.5. Identification of the Extracellular Vesicles (EVs) Proteins

The overlapping of the 71 plasma candidates with the vesicular proteins atlas (HPA) and the Exosome protein atlas (Exocarta) respectively allowed identifying several proteins likely to be secreted through EVs pathways. Thus, 13 vesicular proteins (ACTB, ALB, APOE, FASN, FLNA, HSP90AA1, HSPA1B, IGF1R, LDHB, MPO, PGK1, PPARG and YWHAG), two exosomal proteins (LGALS3BP and CFL1), and three proteins identified simultaneously as vesicular proteins and exosomal proteins (GAPDH, HSPA1A, and LDHA). Finally, 18 putative EVs proteins could be detected in the repertoire of candidate plasma tenderness proteins identify in this study.

### 2.6. QTL Investigation

As seen previously, 14 proteins were identified as located in cattle meat QTL for Shear force and/or Tenderness score from the network analysis (Table 2). Moreover, out of the 57 plasma candidates, 10 proteins including ATP2A2 (Chr. 17), HBB (Chr.15), HSP90AA1 (Chr.21), LAMC1 (Chr.22), LDHA (Chr.29), LDHB (Chr.5), PPARG (Chr.22), PVALB (Chr.5) were located in a cattle QTL for Shear force and ACTC1 (Chr.10), TPM1 (Chr.10) located in a cattle QTL for Tenderness score (Table 2).

## 3. Discussion

As a potential rich source of biomarkers, secreted proteins are targeted by biologists for the discovery of biomarkers [65] especially because they reflect various states of the cells at real time under given conditions. More specifically, secreted proteins in plasma are promising for the identification of low invasive biomarkers circulating in the bloodstream. Therefore, we assumed that in silico prediction of the secretome might help us discovering candidate biomarkers for beef tenderness in the plasma. As a first step in the biomarker identification workflow [66], we designed a study based on the review of the literature and the aggregation of molecular data related to meat tenderness. According to Bonnet et al. [67], we performed a computational reconstruction of the secretome putatively linked to tenderness from the aggregated data, and searched for proteins secreted in the plasma. With this approach, we proposed a list of 71 putative plasma proteins to be investigated further as candidate plasma biomarkers for meat tenderness. Four other plasma candidates from recent literature will thereby expand this list through this discussion. Thus, from this final list of 75 candidate biomarkers, we propose a list of 33 proteins, which are particularly promising for meat tenderness (Table 6). 

### 3.1. Relevance of the Aggregated Dataset

Over the last two decades, 44 studies meeting our criteria of inclusion have identified genetic markers, and proteins or transcripts of which the abundance was related to tenderness. Some of them were proposed as muscle-derived biomarkers for meat quality [68]. These studies corresponded to less than 10% of the curated articles on meat tenderness. From this corpus, we aggregated a full compiled Atlas comprising 469 unique Gene Names, which we considered sufficient for further information mining. From this non-exhaustive dataset, we were able to identify 71 plasma candidate biomarkers for beef tenderness. Moreover, by comparison of the full compiled Atlas with the 67 proteins proposed recently in Picard et al. [68], four additional proteins (COL4A1, HSPA5, ORM1, PDIA3), both predicted as secreted proteins (with Signal-*p* and no TM) and found in the BPA, were included in our list of candidate biomarkers for meat beef tenderness. Thus, these results allowed to enrich, to 75 candidate plasma proteins, the list of candidates proposed in this study. The relevance of the list is supported by the good overview of tenderness mechanisms permitted by the data, as illustrated by GO term enrichment and their semantic analysis. The main pathways involved in meat tenderness (reviewed in [21,33]) were detected with our dataset as illustrated by the top 50 BP terms retrieved by a GO analysis (Table 3). Indeed, we report Biological Processes related to muscle structure and contraction (protein stabilization, muscle contraction and development, chaperone-mediated protein complex assembly, cell adhesion), muscle energy metabolism (gluconeogenesis, glycolytic process, oxidation-reduction process, carbohydrate metabolism), “post-mortem proteolysis” (aging, apoptotic process), “oxidative stress and HSP proteins” (cell detoxification, response to hydrogen peroxide, response to oxidative stress), and “metabolism, transport and cell signalling” (protein folding). The validation of the relevance of the aggregated dataset was a critical step prior to further computational analysis. 

### 3.2. Reconstruction of the Secretome Linked to Tenderness and Identification of Secreted Proteins in Plasma

We propose for the first time a repertoire of secreted proteins related to tenderness. As predicted by bioinformatics, these proteins could be secreted through different pathways.

#### 3.2.1. Proteins Predicted to Be Secreted through Conventional and Unconventional Pathways of Secretion (UPS)

From the aggregated dataset, 11.5 % of the proteins were predicted as secreted proteins through conventional- and 7.7% through alternative pathways. This is consistent with the report that 10–15 % of the human proteome is likely to be secreted through conventional and UPS secretory pathways [69,70]. However, although the bioinformatics reconstruction of the secretome with ProteINSIDE could identify secreted protein thanks to prediction algorithms, it did not enable to distinguish between proteins secreted into the surrounded extracellular fluid and proteins secreted into the bloodstream [67] Noteworthy, by overlapping the repertoire of predicted secreted proteins with a curated non-exhaustive bovine plasma atlas, we depicted 24% of them as putative plasma proteins. This result fits with the report by [71] that 31% of the secreted proteins of the human proteome are found in the plasma. However, the lower proportion of the secreted proteins in plasma in our dataset may be explained by the fact that our plasma atlas was very less that the 10,000 human proteins detected in serum/plasma curated from >500 published studies [70]. This suggests that by using a more complete plasma bovine atlas, we would increase by many the repertoire of secreted proteins in plasma. The semantic analysis of the enriched GO Biological Process associated with the repertoire of predicted secreted proteins in plasma (Table 4) revealed 6 associated biological pathways, linked to “cell adhesion”, “apoptotic process”, “endocytosis”, “response to oxidative stress”, “hydrogen peroxide metabolism”, and “lipid metabolism”. The most canonical pathways associated with the repertoire of 44 proteins were “homeostasis”, “signal transduction (receptor tyrosine kinase signaling, and NR1H2/H3 mediated signaling)”, “immune system (neutrophil degranulation)” and “transport of small molecules (plasma lipoprotein assembly, remodeling, ABC transporter ion channel, mitochondrial calcium ion transport…). These results are in accordance with the literature relating to mechanism involved in non-vesicular UPS secretion [72]; such as “ABC transporter” reported as involved in the maintain of a stable physiological state and homeostasis in vertebrates [73]. Also, the liver X receptors LXR-*α* (NR1H3) and LXR-*β* (NR1H2), a subclass of nuclear receptors, were reported to bind the oxidized forms of cholesterol (or oxysterols), and activate the target gene expression [74]. These observations, suggest that lipid metabolism [75] and by consequence, in the light of our results, the secretion of proteins associated with lipid metabolism (conventional and UPS), could be involved in the tenderness. This is consistent with previous studies linking the lipid metabolism with the meat quality attributes flavour and tenderness [76,77].

#### 3.2.2. Proteins Hypothetically Secreted in the Plasma

By overlapping the repertoire of proteins not hallmarked for secretion (i.e., without a signal P, Target P, or a GO term “secretion”) with the bovine protein atlas, we retrieved proteins known to be found in the plasma. We therefore declared them as proteins hypothetically secreted in the plasma. The biological processes associated with these proteins were associated mainly with muscle contraction, protein stabilization, protein folding, chaperones, carbohydrate metabolism, and endocytosis. Moreover, six BP terms (four related to oxidant status, one to neutrophil degranulation and one to receptor-mediated endocytosis) were shared between the repertoire of secreted proteins in plasma and of hypothetically secreted proteins in plasma. While anti-oxidant proteins (PRDX6, MPO, and ATP2A2) were rather associated with the predicted proteins secreted the former, heat-shock proteins (HSPA1A, HSPA1B, HSP90AA1) were associated with the proteins hypothetically secreted in plasma. The most canonical pathways associated with the repertoire of 13 proteins included “cell-cell communication”, “homeostasis”, “muscle contraction”, “metabolism of proteins”, “metabolism of lipids (citric acid cycle and carbohydrate metabolism)”, “programmed cell death”, “cellular responses to external stimuli”, “organelle biogenesis and maintenance (cilium assembly)”, “autophagy”, “extracellular matrix organization”. Interestingly the primary cilia were described as involved in various pathways related to development and tissue homeostasis, such as *Wnt* [78] or *Hedgehog* [79] pathways. The muscle stem cells need a primary cilium for effective muscle regeneration [80]. The primary cilia were also reported as involved in other vesicular UPS [81].

### 3.3. Extracellular Vesicle Proteins as a Sub Repertoire of Tenderness Proteins Secreted in Plasma

During the last decade, extracellular vesicles (EVs) released by the cells have been described as key actors in intercellular communication in physiological conditions (e.g., heart and muscle development, angiogenesis) [82,83] and in pathogenesis especially in cancer [84]. The EVs are lipid bilayer particles composed of a range of different lipids and proteins (especially phospholipids, cholesterol and tetraspanin proteins), that can carry proteins, RNA and DNA in their aqueous core. EVs include microvesicles (MVs; 100–1000 nm size) or exosomes (30–100 nm size) and apoptotic bodies (1-5 µm) transporting proteins, mRNA, miRNA and lipids in the extracellular medium of cells and putatively in plasma because according to [85,86] all the bio-fluids (e.g., blood, urine, salive, lymphe, milk) contain EVs. Extracellular vesicles represent a potential source for biomarker discovery and can be used for drug and vaccine delivery conditions [87]. EVs are be considered as integrators of tissue physiology and whole-body homeostasis [88,89] EVs secretion is induced in response to extracellular signals such as ATP, interleukins, depolarization, thrombin receptor activation or by cell stress [90,91] Exosome secretion meanwhile can be induced by stress condition, micronutrient starvation, infection or cancer [92]. Recent studies have shown that skeletal muscle is also able to release EVs into the extracellular space [93,94] and to crosstalk with tissues and organs through this mechanism. In this study, we looked whether the hypothetically secreted proteins in plasma could be mapped to EVs. Supporting this hypothesis, we found that 36 % of the proteins were found in an atlas of vesicular proteins and 11 % in the exosome atlas. Therefore, we propose for the first time that EVs and exosome may be a possible reservoir of biomarkers for tenderness. We have identified 13 EVs proteins and two exosomal proteins in the dataset of hypothetically secreted proteins in plasma. Unexpectedly, we also found three vesicular proteins and two exosomal proteins (including the GAPDH protein in common) in the dataset of conventionally and unconventionally secreted proteins in plasma. Similarly, [70] also reported that proteins containing signal peptides that are secreted by the ER-Golgi pathway are also detected in extracellular vesicles. They suggested an unknown mechanism of sorting secreted proteins into these vesicles. Chauhan et al. [95] showed that the GAPDH protein is trafficked to the plasma membrane to be released in the extracellular matrix without use of the classic endoplasmic-Golgi secretion pathway but exosomes and secretory lysosomes.

To our knowledge, the association of EVs or exosomes with tenderness has never been reported. The biological significance of EVs tenderness proteins is unknown but their circulating level in the bloodstream could be a signature of the meat potential of the animals. Regarding their role, recent studies have suggested a role for EVs for the sharing of metabolites and other material between cells or tissues. According to Stahl et al. [96], EVs could operate as “independent metabolic units” that shuttle important molecules (enzymes, metabolites) for muscle homeostasis. Thus, we cannot exclude a role for EVs in unfavorable conditions especially following death of the animal (anoxia, pH and calcium release. The acid environment in muscle fibres after the animal death could promote the release of exosomes by muscle cells [97]. By delivering enzymes and/or metabolites involved in the glycolytic metabolism (e.g., LDHB and PGK1) to muscle cells *post-mortem*, the exosomes could compensate the early stop of glycolytic flux (glycolysis) independently of glycogen availability. EVs could also modulate the redox metabolism (myeloperoxidase (MPO), Thioredoxin-dependent peroxide reductase (PRDX3)) or address some HSP to delivery sites where they could play a crucial role in protecting the cells following death. Indeed, some HSP proteins such as the HSP70 [98,99] were reported in association with the membranes of EVs. More specifically, the HSP90 protein has been described as being exported via exosome vesicles [100,101].

Following network analysis we could include five new proteins found in plasma (CFL1, GC, PLEC, SLC4A1 and VCL) in the repertoire of tenderness hypothetically secreted proteins in plasma. These proteins have not been linked to tenderness so far, but at the exception of GC (vitamin D binding protein), they can be related to known pathways important for meat tenderization. The Cofilin 1, non-muscle (CFL1) is known to be involved in promoting actin polymerisation and organisation of actin filament, lipid metabolism, gene regulation and apoptosis [102]. This protein was also reported as associated with muscle lipid composition [103] Jia et al. [104] compared the *post-mortem* evolution of the proteome muscles differing in their tenderness (the *Longissimus thoracis* (tender) muscle and *Semitendinosus* (tougher) muscle). They reported a decrease in the levels of CFL1. The plectin (PLEC) and the vinculin (VCL) are two major structural components of the muscle cytoskeleton [105] located at the Z-discs [106]. These proteins are important proteins found in the costamere (which attaches myofibrils to the sarcolemma) that are essential for muscle fibre integrity and function (reviewed in [107]). Their proteolytic degradation *post mortem* leads to the disruption of the myofibrillar structure and to tenderisation of the meat. The SLC4A1 gene encodes the Cl^−^/HCO3^−^ anion exchanger 1, an acid loader that exchange one Cl^−^ into cells for onw HCO_3_^−^ out of cells, and thus is involved in the regulation of intracellular pH, especially in erythrocytes and kidney cells [108].

#### Relevance of the Secreted Proteins in Plasma for Tenderness Biomarkers Studies

Thanks to the bioinformatics prediction, we identified 75 proteins related to tenderness putatively secreted in plasma, through conventional, UPS or other pathways including EVs and/or exosome. Consistently, we detected four of them (ACTB, ENO3, GAPDH and MYH7) as differential according to tenderness in a proteomic analysis of the plasma in beef heifers (Boudon, et al., submitted). Twenty-four of the 75 putative plasma proteins (ATP2A2, ACTC1, HBB, HSP90AA1, LAMC1, LDHA, LDHB, PPARG, PVALB, TPM1, CASP8AP2, ACTN1, CAT, CCNB2, CFL1, GSS, MAPK1, NEFL, PRKACB, PSMA7, USP8, YWHAB, YWHAZ and ZBTB21) were encoded by genes located in a bovine meat QTL (shear force or tenderness score). More specifically, six proteins (HSP90AA1, LDHA, LDHB, PPARG, CAT and ORM1) among the 23 putative EVs and/or exosomal proteins were encoded by genes located in a bovine QTL for shear force. Likewise, the 14 plasma proteins identified by network analysis (CASP8AP2, ACTN1, CAT, CCNB2, CFL1, GSS, MAPK1, NEFL, PRKACB, PSMA7, USP8, YWHAB, YWHAZ and ZBTB21) was located in a QTL for shear force and/or a QTL for tenderness score in cattle. Interestingly, CFL1 harbors SNPs in its locus related to beef muscle lipid composition [103] These features made these 33 proteins relevant to be explored as plasma biomarkers for meat tenderness (Table 6).

## 4. Materials and Methods

### 4.1. Data Origin and Literature Search Strategy

#### 4.1.1. Review of the Literature

A computational workflow was created (Figure 1) to retrieve the data and aggregate them from available publications reporting meat tenderness. Briefly, we collected publications on meat tenderness by literature boolean operators: “meat OR beef AND tenderness AND biomarkers”, “meat AND quality” and “muscle AND beef AND proteome (or “transcriptome”, or “genetics”) using MEDLINE (PubMed, https://www.ncbi.nlm.nih.gov/pubmed/), GOOGLE (Google Scholar, https://scholar.google.fr/) and CLAVIRATE (Web Of Science, https://clarivate.com/products/web-of-science/) analytics search until January 2018.

#### 4.1.2. Parameters of Inclusion

All of the articles related to cattle meat tenderness were reviewed and curated based on the relevance and significance of the results. Only, molecular data related to the meat tenderness of *Bos taurus* and *Bos indicus* were conserved. Protein data could come from individual data. Only data with significant correlation of genetic polymorphism with tenderness, or differential abundances of transcripts or proteins according to tenderness as declared by the authors, were kept to build a meat tenderness aggregated dataset. A study associated with one GEO Dataset reporting transcriptomic data was analyzed with GEO2R (https://www.ncbi.nlm.nih.gov/geo/geo2r/) that enabled to compare two groups of samples according to tenderness. The differentially abundant transcripts between tenderness groups were included in our study.

### 4.2. Aggregation of Collected Data

#### 4.2.1. Data Extraction

The molecular data collected from proteomic, transcriptomic or genetic studies were extracted from the articles and aggregated as follows. The proteins identifiers (ID) or gene symbols were retrieved from tables in Portable Document Format (PDF) or from supplementary data files of the publications. Data were extracted with Tabula (www.tabula.technology, Last update 11 February 2017).

#### 4.2.2. Protein Identifiers Standardization

Protein ID and gene symbols were converted into the corresponding Gene Name identifiers (GN), as unique identifiers by use of three tools: Retrieve/ID Mapping tool of the Uniprot database (The UniProt 24), the Protein Identifier Cross-Reference service 25 and/or the ProteCONVERT tool of the ProteINSIDE web interface 26. Last conversion from ID to GN in February 2018.

### 4.3. Gene Ontology

In order to identify biological pathways associated with the aggregated dataset, Gene Ontology (GO) analysis was performed with the ProteINSIDE webservice (http://www.proteinside.org) [109] The GO enrichment analyses were achieved in the Human species in order to extend and promote GO interpretations because the bovine annotations remain limited. Only the Biological Process (BP) were considered. The Benjamini Hochberg (BH) adjusted P-values were considered to establish lists of significant enriched pathways in each dataset as compared to the whole genome. The GO_BP overview was carried out only with annotations with *p*-values < 0.001, minimum of annotated proteins ≥ 2. A table of the GO_BP overview was constructed in a semantic SimRel similarity-based Scatterplots with *p*-values associated to GO terms using REVIGO web tool (http://revigo.irb.hr/) [110] A visualization of the canonical pathways associated with the lists of candidate plasma proteins identified in the study was performed using Reactome tools (https://reactome.org/; voronoi hierarchical representation).

### 4.4. Computational Prediction for the Plasma Secreted Proteins Identification

#### 4.4.1. Prediction of the Secreted Proteins

In order to identify putatively secreted proteins belonging to the aggregated dataset, we used ProteINSIDE, a free web tool (http://www.proteinside.org) [109] that enables retrieving biological information from public databases in a single query. The secretion prediction module of ProteINSIDE runs a local version of SignalP 4.1. From the sequences of input ID proteins, it looks for signal peptide type sequences. The program also checks if proteins are related to a secretory function by looking for GO secretion annotation terms. The aggregated dataset was submitted to a computational prediction of proteins secreted using “custom analysis”, “bovine species”, “signal P” and “increase cleavage site sensitivity (D-cutoff 0.34)” parameters (version of Database 1.2.11, CBS signal-P 4.1 software, May 2018). To declare proteins as “predicted secreted proteins”, we used the following criteria. (1) File tab “Secreted Protein”, Signal-P score > 0.5 and Target-P score ≤ 2 to identify the proteins predicted as secreted through a signal-P sequence and/or a transmembrane domain (TM) (named “conventional predicted secreted proteins”). (2) File tab “other secreted protein”, Target-P score ≤ 3 with GO term associated to identify the proteins predicted as secreted through an unconventional pathway of secretion (or UPS) without signal-P (named “UPS predicted secreted proteins”) [72,111]. The conventional- and UPS- predicted secreted proteins were merged in a single repertoire referred to as predicted secreted proteins. All of the proteins not identified as predicted secreted proteins were “the remaining proteins” (aggregated data minus secreted proteins).

#### 4.4.2. Prediction of Plasma Location

In order to search for the proteins that may be found in the plasma, we compared protein lists using VIB / UGent (http://bioinformatics.psb.ugent.be/webtools/Venn/). The comparisons were performed between the repertoire of predicted secreted proteins and a “Bovine Plasma proteome Atlas” (BPA, *n* = 1101 plasma proteins, which were merged from publications [67] and experimental data (Supplementary Data 3). Similarly, a comparison between the remaining proteins and the BPA was performed to detect hypothetically secreted proteins and found in plasma.

### 4.5. Network Analysis and Protein-Protein Interactions

In order to enrich the list of putative plasma proteins, we used the academic Cytoscape open source software^®^ (Version 3.7.2, https://cytoscape.org/) [112] with the Psicquic plugging web service (https://apps.cytoscape.org/apps/psicquicuniversalclient, up to date, 2017-12-17) [113]. The parameters for network analysis were “MINT database”, “human species”. The proteins that interact with proteins within our dataset were named “interactors”. For representation, the 13 predicted secreted proteins in plasma (conventional and alternative pathways) are shown in purple ellipses. The 44 hypothetically secreted proteins in plasma are shown in pink ellipses. The green rectangle refer to interactor identify using the MINT Cytoscape analysis.

### 4.6. Search for QTL

By using the ProteQTL module of ProteINSIDE, we searched for the location of genes encoding the proteins of interest within published Quantitative trait loci (QTL) for tenderness.

### 4.7. Identification of the EVs Proteins

In order to test the hypothesis that membrane-derived vesicles secretion could be associated with tenderness, we compared the repertoires of candidate proteins with the Human Protein Atlas (HPA) that lists the vesicular proteins experimentally detected in the vesicles (referred to as “vesicular protein Atlas”, *n* = 1998; 2019 October, 28th; https://www.proteinatlas.org/) and the Exosome protein atlas (*n* = 100, 25 October 2019, http://exocarta.org/) that lists the proteins detected in exosomes.

### 4.8. Dataset Descriptors

Four datasets were generated in this study. The aggregated dataset merged from three individual lists related to beef tenderness, namely a “proteomic dataset”, a “transcriptomic dataset”, and a “genetic dataset” The aggregated dataset (named “full compiled atlas”) was deposited as “.xls” files at the French INRA public repository (Portail Data INRA, data.inra.fr) hosted by Dataverse.org and is directly available at [63]. In addition, the two repertoires generated by reconstruction of the secretome were named the “predicted secreted proteins in plasma” and the “proteins hypothetically secreted in plasma”. Finally, the merged of the “predicted secreted proteins in plasma”, the “proteins hypothetically secreted in plasma” and the “interactors” putatively found in the plasma” generated the final list of candidate plasma proteins proposed by this study as putative low-invasive candidates for meat tenderness in beef cattle.

## 5. Conclusions

This study is the first to use data aggregated from a corpus of published data for the purpose of identifying novel meat tenderness in muscle (thanks to PPi) and in the plasma. We propose for the first time a non-exhaustive list of 75 candidate biomarkers for tenderness in the plasma. Combined with QTL data and recent literature, 33 are of particular interest for further evaluation and validation for future low-invasive approach, among which four proteins recently reported as muscle tenderness biomarkers and found in plasma. Another original finding of this study is that the secretion pathway of 13 of these plasma proteins could be the membrane-derived vesicle secretion. The 33 plasma candidate biomarkers for meat tenderness identified in this study require further assessment and validation.

## Figures and Tables

**Table 1:** Description of the computational analysis of the datasets included in the study.

Dataset	Number of ID Gene Names	Predicted Secreted Proteins (Conventional Pathways)	Predicted Secreted Proteins (Unconventional Pathways, or UPS)	Remaining Proteins	Predicted Secreted Proteins (Conventional and UPS) + Plasma	Hypothetically Secreted Proteins + Plasma
**Proteomics** **(26 Articles)**	139	8	8	123	2	27
**Transcriptomics** **(** **11 Articles)**	249	29	18	202	6	19
**Genetics** **(12 Articles)**	123	19	14	90	5	6
**General Bilan (Unique ID)**	511 (469)	56 (54)	40 (36)	415 (379)	13 (13)	52 (44)

The table presents the number of ID Gene Names for each dataset. Predictive secreted proteins (conventional pathways, (i): number of ID Gene Names identified as predicted secreted with signal-P sequence using ProteINSIDE predictive analysis (Signal-P > 0.5; Target-P ≤ 2). Predictive secreted proteins (unconventional pathways (UPS), (ii): number of ID Gene Names identified as predicted secreted without signal-P sequence using ProteINSIDE predictive analysis (Target-P ≤ 3). Remaining proteins: number of ID Gene Names non-predicted as secreted using ProteINSIDE. Predicted secreted proteins (conventional and alternative) in plasma: Number of ID Gene Names: (i) and (ii) found in the plasma by overlapping with the Bovine Proteome Atlas (BPA). Other proteins hypothetically secreted in plasma: Remaining proteins that were found in plasma by overlapping with the Bovine Proteome Atlas (BPA). In brackets: number of unique ID Gene Names associated with each category of proteins in the aggregated dataset. Unconventional pathways of secretion (UPS).

**Table 2:** List of the 71 candidate plasma proteins associated with beef tenderness.

ID Gene Name	QTL	EVs Proteins (HPA, *n* = 1998)	Exosomal Proteins (Exocarta, *n* = 100)
13 predicted secreted proteins + plasma (conventional and UPS)			
*APOE*		X	
*ATP2A2*	Shear force (Ch. 17)		
*CDH13*			
*COL11A1*			
*CUBN*			
*EPHA7*			
*GAPDH*		X	X
*GLG1*			
*LGALS3BP*			X
*MPO*		X	
*PCDH7*			
*PRDX6*			
*TG*			
44 hypothetically secreted proteins + plasma			
*ACTA1*			
*ACTB*		X	
*ACTC1*	Tenderness score (Chr.10)		
*ALB*		X	
*ATP5B*			
*BPGM*			
*CAPN2*			
*CASP8*			
*CCT8*			
*CENPF*			
*CKM*			
*COL13A1*			
*DES*			
*ENO3*			
*FASN*		X	
*FGF12*			
*FLNA*		X	
*GBP1*			
*HBB*	Shear force (Ch.15)		
*HSP90AA1*	Shear force (Chr.21)	X	
*HSPA1A*		X	X
*HSPA1B*		X	
*IGF1R*		X	
*LAMC1*	Shear force (Chr.22)		
*LDHA*	Shear force (Ch.29)	X	X
*LDHB*	Shear force (Ch.5)	X	
*LOX*			
*LRRC16A*			
*MYH2*			
*MYH3*			
*MYH7*			
*NID1*			
*PGAM2*			
*PGK1*		X	
*PPARG*	Shear force (Chr.22)	X	
*PRDX3*			
*PSMB2*			
*PVALB*	Shear force (Chr.5)		
*RGS2*			
*SDHB*			
*TPM1*	Tenderness score (Chr.10)		
*TPM3*			
*TUFM*			
*YWHAG*		X	
14 plasma proteins from Network/QTL			
*CASP8AP2*	Tenderness score (Chr.9)		
*ACTN1*	Tenderness score (Chr.10)		
*CAT*	Shear force (Chr.15)	X	
*CCNB2*	Tenderness score (Chr.10)		
*CFL1*	Tenderness score and Shear force (Chr.29)		X
*GSS*	Shear force (Chr.13)		
*MAPK1*	Shear force (Chr.17)		
*NEFL*	Shear force (Chr.8)		
*PRKACB*	Shear force (Chr.3)		
*PSMA7*	Shear force (Chr.13)		
*USP8*	Tenderness score (Chr.10)		
*YWHAB*	Shear force (Chr.13)		X
*YWHAZ*	Shear force (Chr.14)		X
*ZBTB21*	Shear force (Chr.1)		

We report all the proteins proposed as plasma candidates for beef tenderness: 13 predicted secreted proteins identified using ProteINSIDE tool, 44 hypothetically secreted found by overlapping the repertoire of proteins not hallmarked for secretion with the BPA, and 14 plasma proteins revealed from the network and QTL analysis. EVs: The vesicular proteins were retrieved by overlapping with the Vesicular protein Atlas from HPA. The exosome proteins were retrieved by overlapping with the Exosome proteins from Exocarta Atlas. BPA: Bovine Plasma proteome Atlas. The information on the location of the genes encoding proteins of interest within published QTL for tenderness retrieved using the ProteoQTL module of ProteINSIDE. This module interrogates a publicly available QTL library in Animal QTL database that contains cattle QTL and the published data associated. In brackets in the QTL column: chromosome associated with the Tenderness score and/or Shear force QTL. “X” means that the protein was found in the considered HPA and/or Exocarta atlas.

**Table 3:** TOP50 Gene Ontology terms associated with the 469 proteins of the aggregated dataset related to meat tenderness.

GO Term	Description	ID Gene Name	Enrichment in Dataset (%)	Enrichment in Genome Database (%)	*p*-value Adjusted
**Inflammatory Response**					
GO:0043312	neutrophil degranulation	*GDI2 ASAH1 PNP HSP90AA1 PGM1 PSMC2 PKM MPO PLAC8 HSPA1A PRDX6 PGAM1 CCT8 ALDOA HSPA1B ATP8B4 CLEC12A SERPINA3 HSPA6 GSTP1 HBB HSPA8 DNAJC3 ATP11A* *DGAT1*	6.14	5.17	1.98 × 10^−22^
GO:0042493	response to drug	*ADA CASP3 SOD1 NPPC PPARG LOX ENO3 VAV3 ABCG5 LGALS1 CENPF AQP1 ACTC1 PNP CTNNB1 KCNK3 SST FABP3 LDHA* *LCK*	4.91	5.39	2.53 × 10^−18^
GO:0055085	transmembrane transport	*SLC6A9 ABCA12 VDAC2 CACNA1C SLC25A12 ABCG5 ANKH ITPR1 VDAC1 PSMB2 SLC6A20 HCN1 KCND2 SLCO3A1 SLC39A11 TRPM3 PSMC2 SLC9A9 AQP1 SLC9A7* *SLC25A48*	5.16	2.9	3.38 × 10^−14^
GO:0098869	cellular oxidant detoxification	*PARK7 APOE ALB TXN PRDX3 PRDX6 GSTP1* *HBB*	1.97	50	3.99 × 10^−14^
GO:0042542	response to hydrogen peroxide	*MB SIRT1 LDHA ADA PRDX3 PARK7 CAPN2 HBB CASP3 CRYAB HMOX1 SOD1*	2.95	11.11	1.71 × 10^−14^
GO:0045471	response to ethanol	*GSTP1 MSTN LEP RGS2 CASP8 CA3 NQO1 ACTC1 TUFM NPPC* *SOD1*	2.7	9.32	1.38 × 10^−12^
GO:0071356	cellular response to tumor necrosis factor	*BAG4 SIRT1 GPD1 FABP4 CCL25 MYOD1 ZFP36L1 GBP1 GBP3* *ASAH1*	2.46	9.01	2.31 × 10^−11^
> GO:0071346	cellular response to interferon-gamma	*GBP7 GBP3 CCL25 GBP6 GBP1 GBP5* *GAPDH*	1.72	12.5	5.78 x 10^−9^
GO:0032355	response to estradiol	*LEP GSTP1 CTNNB1 CRYAB OXT PTGFR CASP3 NQO1 CASP8 GHR*	2.46	7.87	7.59 × 10^−11^
GO:0006811	ion transport	*VDAC2 KCND2 ATP2A2 CACNA1C KCNK3 SLCO3A1 HCN1 VDAC1 ITPR1 KCNJ3 CACNA2D1 SLC9A9 SLC9A7 TRPM3 ATP5PD SLC39A11 SCN2B CLCA3P KCNJ15 CHRNE*	4.91	1.87	2.33 × 10^−10^
GO:0034620	cellular response to unfolded protein	*HSPA6 HSPA9 HSPA1A HSPA8 HSPA1B*	1.23	83.33	1.09 × 10^−9^
> GO:0006986	response to unfolded protein	*HSPA9 HSPB1 HSPA1B HSPA8 DNAJC3 DNAJA1 DNAJB5 HSPA6 HSP90AA1 HSPH1 HSPB2 HSPA1A*	2.95	25	3.40 × 10^−18^
GO:1900034	regulation of cellular response to heat	*BAG4 HSPA1B HSPA8 CRYAB SIRT1 HSPA1A HSPH1 HSP90AA1*	1.97	10.26	1.44 × 10^−9^
GO:0032869	cellular response to insulin stimulus	*GCLC PKM PPARG ZFP36L1 GOT1 YWHAG GSTP1 LEP*	1.97	10.13	1.54 × 10^−9^
GO:0009409	response to cold	*CASP8 CXCL10 PPARG METRNL PLAC8 HSP90AA1 ACADVL*	1.72	14.89	2.03 × 10^−9^
> GO:0034605	cellular response to heat	*HSP90AA1 HMOX1 HSPA8 CXCL10 HSPA1B HSPA6 HSPA9 HSPA1A ATP2A2*	2.21	19.57	6.79 × 10^−13^
GO:0001666	response to hypoxia	*CASP3 HMOX1 CRYAB PKM MB ADA NPPC LEP CAPN2 ITPR1 LDHA*	2.7	4.17	3.16 × 10^−9^
GO:0006979	response to oxidative stress	*PRDX6 MPO SGK2 HMOX1 SIRT1 CA3 NQO1 APOE PRDX3 GCLC NDUFB4 SOD1*	2.95	3.48	3.35 × 10^−9^
GO:0006954	inflammatory response	*IDO1 NFATC3 CSRP3 CCR5 CCR3 CCL25 CXCL10 FOLR2 SERPINA3 PTGFR RPS6KA4 PARK7 GBP5*	3.19	2.97	3.66 × 10^−9^
**Gluconeogenesis**					
GO:0055114	oxidation-reduction process	*PTGR1 LDHB GAPDH NDUFV2 SOD1 TXN PRDX6 NDUFB4 HGD VAT1L LOX NDUFS3 NDUFV1 MDH1 MDH2 ME2 ALDH2 LDHA UQCRC1 MPO NQO1 ACADVL BCKDHB PDHB NDUFS1 DMGDH IDH3A NDUFA10 SOD2 WWOX UQCRH IDO1 PRDX3 HMOX1 ALDH1B1 SDHB GPD1 FASN*	9.34	8.48	2.82 × 10^−41^
GO:0006094	gluconeogenesis	*ENO1 ENO3 PGAM2 SLC25A12 GOT1 TPI1 MDH1 PGAM1 PGM1 PGK1 SDS GPD1 GAPDH ALDOA MDH2*	3.69	34.09	2.02 × 10^−24^
GO:0061621	canonical glycolysis	*PKM ENO1 PGAM1 TPI1 PGAM2 BPGM PGK1 PFKM ENO3 ALDOA GAPDH*	2.7	40.74	1.40 × 10^−18^
GO:0046034	ATP metabolic process	*MYH4 MYH7 ATP5PD NDUFS1 MYH8 HSPA1B HSPA1A ATP5B ENPP3 MYH3 AK1 HSPA8*	2.95	10.26	3.83 × 10^−14^
> GO:0006096	glycolytic process	*PGM1 PRKAG3 GAPDH PGK1 ENO1 BPGM PGAM1 PFKM PKM PGAM2 ALDOA ENO3 TPI1 LDHA*	3.44	35.9	4.62 × 10^−23^
GO:0006099	tricarboxylic acid cycle	*IDH3A DLST ME2 PDHB IREB2 MDH2 MDH1 SDHB*	1.97	26.67	2.12 × 10^−12^
**Protein Stabilization**					
GO:0050821	protein stabilization	*HSPA1A GAPDH PFN1 PARK7 SAXO1 PHB HSP90AA1 HSPA1B CRYAB CCT8 PPIB FLNA*	2.95	7.89	6.22 × 10^−13^
GO:0045944	positive regulation of transcription from RNA polymerase II promoter	*NFATC3 EBF1 PARK7 RPS6KA4 MYOD1 CSRP3 SMAD1 PLAC8 SOX5 SIRT1 MYT1 TBX15 WWOX PAX7 NLRC5 CTNNB1 CDH13 CXCL10 PFKM PPARG SIM1*	5.16	2.22	3.70 × 10^−12^
> GO:0000122	negative regulation of transcription from RNA polymerase II promoter	*PPARG WWOX DNAJB5 TBX15 LEP PHB CUX2 CXXC5 AURKB TENM2 STRAP EHMT1 SIRT1 CTNNB1 COPS2 TXN RORC ENO1*	4.42	2.65	1.15 × 10^−11^
GO:1904706	negative regulation of vascular smooth muscle cell proliferation	*HMOX1 GSTP1 PPARG TPM1 SOD2*	1.23	55.56	4.17 × 10^−9^
GO:0008285	negative regulation of cell proliferation	*SPRY1 CTNNB1 CGREF1 CDH13 NPPC PPARG FABP3 SOD2 SST HMOX1 HSPA1A PTPRK PHB HSPA1B CLDN19*	3.69	2.29	4.81 × 10^−9^
GO:0030308	negative regulation of cell growth	*NDUFS3 ENO1 CRYAB HSPA1B PHB HSPA1A SIRT1 MYL2 PPARG APBB2*	2.46	6.25	5.75 × 10^−10^
GO:0046716	muscle cell cellular homeostasis	*PFKM CFL2 ALDOA MSTN SOD1 LOX*	1.47	31.58	9.74 × 10^−10^
**Chaperone-Mediated Protein Complex Assembly**					
GO:0051085	chaperone mediated protein folding requiring cofactor	*HSPA1B HSPA9 HSPA8 HSPH1 HSPA1A DNAJB5 HSPA6*	1.72	53.85	1.52 × 10^−12^
GO:0042026	protein refolding	*PPIB HSPA8 HSPA6 HSPA1A HSPA1B HSPA9 HSP90AA1*	1.72	33.33	1.94 × 10^−11^
**Carbohydrate Metabolism**					
GO:0005975	carbohydrate metabolic process	*PYGM ALDH2 PGM1 LCT PDK4 GPD1 MDH1 LDHB LDHA ALDH1B1 BPGM POFUT2 PDHB MDH2 IDH3A*	3.69	3.25	5.93 × 10^−11^
**Aging**					
GO:0045214	sarcomere organization	*TPM1 FHOD3 WDR1 TNNT1 MYH3 TNNT3 CFL2 KLHL41 CSRP3*	2.21	23.68	1.59 × 10^−13^
> GO:0007517	muscle organ development	*MYOD1 CSRP3 PAX7 CRYAB MYH3 FHL3 CENPF CXCL10 MSTN SIRT1*	2.46	9.62	1.31 × 10^−11^
GO:0007568	aging	*PBEF1 GCLC ENO3 AURKB SOD1 CNP CRYAB CTNNA1 ADA MPO NQO1*	2.7	4.45	1.67 × 10^−9^
**Muscle Contraction and Development**					
GO:0006936	muscle contraction	*CHRNE CRYAB DES MYH8 MYH1 TNNT3 MYL6B MYH7 MYH4 TNNI2 ACTA1 MYLPF TNNT1 MYL1 MYH2 TPM1 TPM3 CKMT2*	4.42	8.45	1.09 × 10^−19^
> GO:0003009	skeletal muscle contraction	*MYH3 TNNT1 TNNI2 MYH8 ATP2A2 MYH7 TNNT3*	1.72	25.93	8.00 × 10^−11^
> GO:0030049	muscle filament sliding	*DES MYL3 TPM1 TNNT1 MYL1 MYH3 TNNT3 MYL2 ACTN3 MYH8 MYH4 MYH2 TPM3 ACTC1 MYH7 ACTA1 TNNI2 MYL6B*	4.42	47.37	2.23 × 10^−31^
> GO:0060048	cardiac muscle contraction	*CSRP3 TPM1 TNNT3 MYL1 TNNT1 MYH7 TNNI2 MYL2 MYL3 SCN2B ACTC1*	2.7	24.44	2.28 × 10^−16^
GO:0007275	multicellular organism development	*NFATC3 TAPT1 SEMA3E COL13A1 RECQL4 SIM1 SIRT1 TNP1 EBF1 SPRY1 PRRX2 PPARG MYOD1 CSRP3 LRP4 CENPF PAX7 ZFP36L1 MYT1 RORC CYLC1 EPHA7 TPI1*	5.65	3.36	8.30 × 10^−17^
> GO:0007507	heart development	*FGF12 PPARG CASP3 RBM20 CACNA1C CTNNB1 OXT LOX ZFP36L1 MB MYL2 CSRP3*	2.95	6.86	2.74 × 10^−12^
**Cell Adhesion**					
GO:0007155	cell adhesion	*TROAP NID1 CTNNA3 TENM2 LYVE1 NTM CCR3 LAMA3 ADA CTNNA1 CDH13 MYBPH ATP2A2 CGREF1 COL13A1 PCDH7 LAMC1 MPDZ PTPRK DDR2 DSCAML1 LGALS3BP CTNNB1*	5.65	2.74	5.07 × 10^−15^
**Protein Folding**					
GO:0006457	protein folding	*HSPA9 DNAJA1 CRYAB NPPC CCT8 PPIB HSP90AA1 DNAJB11 DNAJB5 BAG4 HSPA8*	2.7	4.85	7.57 × 10^−10^
**Apoptotic Process**					
GO:0006915	apoptotic process	*SHC4 TMEM14A ZFP36L1 PRDX3 AVEN BCL2L14 GAPDH NSG1 EPHA7 LGALS1 CASP3 CASP8 HMOX1 SIRT1 ITPR1 HINT1 VDAC1 WWOX*	4.42	3.01	1.67 × 10^−12^
> GO:0043066	negative regulation of apoptotic process	*CTNNB1 DNAJC3 HSPB1 NQO1 AQP1 TMEM14A ACTC1 HSPA9 HSPA1B MPO GSTP1 AVEN GCLC SOD1 PARK7 CRYAB ADA IGF1R CASP3 BAG4 PLAC8 SIRT1 DNAJA1 PTGFR PKHD1 HSPA1A CTNNA1 ALB FLNA PRDX3 PAX7 LEP APBB2*	8.11	4.02	3.58 × 10^−26^

We report the Top5O of the “Biological process” Gene Ontology terms identified with a significant *p*-value (*p*-value < 0.001) and associated with a minimum of two proteins. This GO Table was obtained using REVIGO (semantic SimRel measure) including GO terms and *p*-value parameters. ID Gene Name: Proteins identified as related with tenderness within each Gene Ontology group. Enrichment in Dataset (%): Percentage of enrichment within the dataset. Enrichment in genome Database (%): Percentage of enrichment without the genome Database used by the ProteINSIDE algorithm analysis. (“>” GO term): GO term included in up-GO term by removing redundant GO terms.

**Table 4:** Gene Ontology of the 13 predicted secreted proteins in plasma.

GO Term	Description	ID Gene Name	Enrichment in Dataset (%)	Enrichment in Genome Database (%)	*p*-Value Adjusted
**Cell Adhesion**					
GO:0007155	cell adhesion	*PCDH7 LGALS3BP ATP2A2 CDH13*	30.77	0.48	4.00 × 10^−5^
> GO:0007156	homophilic cell adhesion via plasma membrane adhesion molecules	*CDH13 PCDH7*	15.38	1.28	1.17 × 10^−3^
**Apoptotic Process**					
GO:0006874	cellular calcium ion homeostasis	*APOE ATP2A2*	15.38	0.55	1.98 × 10^−3^
> GO:0045454	cell redox homeostasis	*PRDX6 MPO*	15.38	2.78	3.38 × 10^−4^
GO:0006915	apoptotic process	*EPHA7 GAPDH*	15.38	0.33	3.04 × 10^−3^
**Endocytosis**					
GO:0002576	platelet degranulation	*PCDH7 LGALS3BP*	15.38	1.63	7.81 × 10^−4^
> GO:0043312	neutrophil degranulation	*MPO PRDX6*	15.38	0.41	2.54 × 10^−3^
GO:0034599	cellular response to oxidative stress	*ATP2A2 PRDX6*	15.38	1.03	1.32 × 10^−3^
GO:0006898	receptor-mediated endocytosis	*APOE CUBN*	15.38	0.86	1.44 × 10^-3^
GO:0006897	endocytosis	*LGALS3BP CUBN*	15.38	0.49	2.19 × 10^−3^
**Response to Oxidative Stress**					
GO:0098869	cellular oxidant detoxification	*PRDX6 APOE*	15.38	12.5	3.70 × 10^−5^
GO:0006979	response to oxidative stress	*APOE MPO PRDX6*	23.08	0.87	9.90 × 10^−5^
GO:0050832	defense response to fungus	*MPO GAPDH*	15.38	4.44	1.54 × 10^−4^
GO:0055114	oxidation-reduction process	*MPO GAPDH PRDX6*	23.08	0.67	1.78 × 10^−4^
**Hydrogen Peroxide Metabolism**					
GO:0042744	hydrogen peroxide catabolic process	*PRDX6 MPO*	15.38	10.53	4.40 × 10^−5^
**Lipid Metabolism**					
GO:0034384	high-density lipoprotein particle clearance	*CUBN APOE*	15.38	22.22	2.20 × 10^−5^
> GO:0034374	low-density lipoprotein particle remodeling	*APOE MPO*	15.38	15.38	3.00 x 10^−5^
GO:0008203	cholesterol metabolic process	*CUBN APOE*	15.38	1.82	6.70 × 10^−4^
GO:0006629	lipid metabolic process	*APOE CUBN PRDX6*	23.08	0.27	1.32 × 10^−3^
GO:0008202	steroid metabolic process	*CUBN APOE*	15.38	0.8	1.44 × 10^−3^
GO:0032496	response to lipopolysaccharide	*MPO ATP2A2*	15.38	0.72	1.56 × 10^−3^

We report all of the “Biological Process” terms associated with the Gene Ontology annotations identified with significant *p*-values (*p*-value < 0.05) and associated with minimum of two proteins. This GO Table was obtained using REVIGO (semantic SimRel measure) including GO terms and *p*-value parameters. ID Gene Name: Proteins identified as related with tenderness within each Gene Ontology group. Enrichment in Dataset (%): Percentage of enrichment within the dataset. Enrichment in genome Database (%): Percentage of enrichment without the genome Database used by the ProteINSIDE algorithm analysis. (“>” GO term): GO term included in up-GO term by removing redundant GO terms.

**Table 5:** Gene Ontology of the 44 hypothetically secreted proteins in plasma.

GO Term	Function	*ID Gene Name*	Enrichment in Dataset (%)	Enrichment in Genome Database (%)	*p*-Value Adjusted
**Muscle Contraction, Structure and Development**					
GO:0030049	muscle filament sliding	*DES ACTC1 ACTA1 MYH7 TPM3 TPM1 MYH3 MYH2*	18.18	21.05	1.80 × 10^−18^
GO:0006936	muscle contraction	*MYH2 TPM3 ACTA1 DES TPM1 MYH7*	13.64	2.82	2.32 × 10^−9^
GO:0050821	protein stabilization	*HSPA1B CCT8 FLNA HSPA1A HSP90AA1*	11.36	3.29	3.27 × 10^−8^
GO:0090063	positive regulation of microtubule nucleation	*HSPA1A HSPA1B*	4.55	50	1.40 × 10^−5^
GO:0030240	skeletal muscle thin filament assembly	*ACTC1 ACTA1*	4.55	40	1.82 × 10^−5^
GO:0030198	extracellular matrix organization	*LAMC1 COL13A1 LOX NID1*	9.09	1.34	1.97 × 10^−5^
GO:0007507	heart development	*LOX PPARG FGF12*	6.82	1.71	1.43 × 10^−4^
GO:0007015	actin filament organization	*TPM3 TPM1 ACTC1*	6.82	1.54	1.87 × 10^−4^
GO:0003009	skeletal muscle contraction	*MYH7 MYH3*	4.55	7.41	2.22 × 10^−4^
GO:0045214	sarcomere organization	*MYH3 TPM1*	4.55	5.26	3.97 × 10^−4^
GO:0021762	substantia nigra development	*LDHA ACTB*	4.55	4.76	4.57 × 10^−4^
GO:0055010	ventricular cardiac muscle tissue morphogenesis	*MYH7 TPM1*	4.55	4.26	5.43 × 10^−4^
**Muscle Energy Metabolism**					
GO:0006096	glycolytic process	*PGAM2 LDHA PGK1 BPGM ENO3*	11.36	12.82	8.64 × 10^−11^
GO:0061621	canonical glycolysis	*PGAM2 BPGM PGK1 ENO3*	9.09	14.81	6.04 × 10^−9^
GO:0046034	ATP metabolic process	*HSPA1A MYH3 HSPA1B MYH7 ATP5B*	11.36	4.27	1.02 × 10^−8^
GO:0055114	oxidation-reduction process	*LDHA LDHB FASN SDHB LOX PRDX3*	13.64	1.34	1.25 × 10^−7^
GO:0006094	gluconeogenesis	*PGK1 ENO3 PGAM2*	6.82	6.82	4.83 × 10^−6^
GO:0060048	cardiac muscle contraction	*MYH7 TPM1 ACTC1*	6.82	6.67	5.06 × 10^−6^
**Apoptosis, Death Cell and Proteolysis**					
GO:0043066	negative regulation of apoptotic process	*HSPA1A FLNA ALB IGF1R PRDX3 HSPA1B ACTC1*	15.91	0.85	1.38 × 10^−7^
GO:1903265	positive regulation of tumor necrosis factor-mediated signaling pathway	*HSPA1A HSPA1B*	4.55	33.33	2.19 × 10^−5^
GO:0038096	Fc-gamma receptor signaling pathway involved in phagocytosis	*ACTB MYH2 HSP90AA1*	6.82	2.27	7.01 × 10^−5^
GO:1900740	positive regulation of protein insertion into mitochondrial membrane involved in apoptotic signaling pathway	*CASP8 YWHAG*	4.55	6.67	2.67 × 10^−4^
GO:0006898	receptor-mediated endocytosis	*ALB HSP90AA1 HBB*	6.82	1.29	2.99 × 10^−4^
GO:2001240	negative regulation of extrinsic apoptotic signaling pathway in absence of ligand	*HSPA1B HSPA1A*	4.55	5.71	3.46 × 10^−4^
GO:0032757	positive regulation of interleukin-8 production	*HSPA1A HSPA1B*	4.55	4.44	5.11 × 10^−4^
**Oxidative Stress and HSP Proteins**					
GO:0098869	cellular oxidant detoxification	*PRDX3 ALB HBB*	6.82	18.75	4.68 × 10^−7^
GO:0042542	response to hydrogen peroxide	*LDHA HBB CAPN2 PRDX3*	9.09	3.7	7.05 × 10^−7^
GO:0090084	negative regulation of inclusion body assembly	*HSPA1A HSPA1B*	4.55	18.18	5.46 × 10^−5^
GO:0042744	hydrogen peroxide catabolic process	*PRDX3 HBB*	4.55	10.53	1.25 × 10^−4^
GO:0034599	cellular response to oxidative stress	*HSPA1B HSPA1A PRDX3*	6.82	1.55	1.86 × 10^−4^
GO:0045429	positive regulation of nitric oxide biosynthetic process	*HBB HSP90AA1*	4.55	4.88	4.40 × 10^−4^
**Metabolism, Transport and Cell Signaling**					
GO:0042493	response to drug	*PPARG CENPF LDHA LOX ACTC1 ENO3*	13.64	1.62	4.29 × 10^−8^
GO:0042026	protein refolding	*HSPA1A HSP90AA1 HSPA1B*	6.82	14.29	8.17 × 10^−7^
GO:0045471	response to ethanol	*RGS2 ACTC1 CASP8 TUFM*	9.09	3.39	8.78 × 10^−7^
GO:0034605	cellular response to heat	*HSPA1A HSPA1B HSP90AA1*	6.82	6.52	5.30 × 10^−6^
GO:0009409	response to cold	*HSP90AA1 PPARG CASP8*	6.82	6.38	5.55 × 10^−6^
GO:0006986	response to unfolded protein	*HSPA1B HSP90AA1 HSPA1A*	6.82	6.25	5.80 × 10^−6^
GO:0070370	cellular heat acclimation	*HSPA1B HSPA1A*	4.55	66.67	9.89 × 10^−6^
GO:0070434	positive regulation of nucleotide-binding oligomerization domain containing 2 signaling pathway	*HSPA1B HSPA1A*	4.55	66.67	9.89 × 10^−6^
GO:0090131	mesenchyme migration	*ACTC1 ACTA1*	4.55	40	1.82 × 10^−5^
GO:1900034	regulation of cellular response to heat	*HSPA1A HSPA1B HSP90AA1*	6.82	3.85	1.88 × 10^−5^
GO:0034620	cellular response to unfolded protein	*HSPA1A HSPA1B*	4.55	33.33	2.19 × 10^−5^
GO:0010389	regulation of G2/M transition of mitotic cell cycle	*YWHAG CENPF HSP90AA1*	6.82	2.56	5.37 × 10^−5^
GO:0051085	chaperone cofactor-dependent protein refolding	*HSPA1A HSPA1B*	4.55	15.38	7.01 × 10^−5^
GO:0051092	positive regulation of NF-kappaB transcription factor activity	*PRDX3 HSPA1B HSPA1A*	6.82	2.24	7.18 × 10^−5^
GO:1901673	regulation of mitotic spindle assembly	*HSPA1A HSPA1B*	4.55	13.33	8.55 × 10^−5^
GO:0051131	chaperone-mediated protein complex assembly	*HSPA1A HSP90AA1*	4.55	12.5	9.52 × 10^−5^
GO:0030308	negative regulation of cell growth	*HSPA1A HSPA1B PPARG*	6.82	1.88	1.14 × 10^−4^
GO:0046718	viral entry into host cell	*HSPA1A HSPA1B*	4.55	9.09	1.61 × 10^−4^
GO:0031396	regulation of protein ubiquitination	*HSPA1A HSPA1B HSP90AA1*	6.82	1.14	4.03 × 10^−4^
GO:0001895	retina homeostasis	*ACTB ALB*	4.55	5	4.23 × 10^−4^
GO:0046677	response to antibiotic	*CASP8 HSP90AA1*	4.55	4.08	5.85 × 10^−4^
**Immune System and Blood Coagulation**					
GO:0070527	platelet aggregation	*HBB FLNA ACTB*	6.82	7.14	4.46 × 10^−6^
GO:0043312	neutrophil degranulation	*HBB HSPA1B HSP90AA1 CCT8 HSPA1A*	11.36	1.03	4.83 × 10^−6^
GO:1904706	negative regulation of vascular smooth muscle cell proliferation	*PPARG TPM1*	4.55	22.22	4.11 × 10^−5^
GO:0030224	monocyte differentiation	*FASN PPARG*	4.55	11.76	1.05 × 10^−4^
GO:0045648	positive regulation of erythrocyte differentiation	*HSPA1B HSPA1A*	4.55	8.7	1.72 × 10^−4^

We report all of the Biological Process associated with the Gene Ontology annotations identified with a significant *p*-values (*p*-value < 0.001) and associated with minimum of two proteins. This GO Table was obtained using REVIGO (semantic SimRel measure) including GO terms and *p*-value parameters. ID Gene Name: Proteins identified as related with tenderness within each Gene Ontology group. Enrichment in Dataset (%): Percentage of enrichment within the dataset. Enrichment in genome Database (%): Percentage of enrichment without the genome Database used by the ProteINSIDE algorithm analysis. (“>” GO term): GO term included in up-GO term by removing redundant GO terms.

**Table 6:** List of the 33 promising plasma biomarkers associated with beef tenderness identified in this study.

ID Gene Name	QTL	Overlapping (Picard & Gagaoua 2019)	Promising Candidates
31 plasma candidate biomarkers identify through this study			
*ATP2A2*	Shear force (Ch. 17)		X
*GAPDH*		X	X
*ACTA1*		X	X
*ACTC1*	Tenderness score (Chr.10)		X
*ALB*		X	X
*ENO3*		X	X
*HBB*	Shear force (Ch.15)		X
*HSP90AA1*	Shear force (Chr.21)		X
*LAMC1*	Shear force (Chr.22)		X
*LDHA*	Shear force (Ch.29)		X
*LDHB*	Shear force (Ch.5)		X
*MYH7*		X	X
*PPARG*	Shear force (Chr.22)		X
*PVALB*	Shear force (Chr.5)		X
*TPM1*	Tenderness score (Chr.10)		X
*CASP8AP2*	Tenderness score (Chr.9)		X
*ACTN1*	Tenderness score (Chr.10)		X
*CAT*	Shear force (Chr.15)		X
*CCNB2*	Tenderness score (Chr.10)		X
*CFL1*	Tenderness score and Shear force (Chr.29)		X
*GSS*	Shear force (Chr.13)		X
*MAPK1*	Shear force (Chr.17)		X
*NEFL*	Shear force (Chr.8)		X
*PRKACB*	Shear force (Chr.3)		X
*PSMA7*	Shear force (Chr.13)		X
*USP8*	Tenderness score (Chr.10)		X
*YWHAB*	Shear force (Chr.13)		X
*YWHAZ*	Shear force (Chr.14)		X
*ZBTB21*	Shear force (Chr.1)		X
4 putative plasma candidates identify from Picard and Gagaoua, 2020			
*COL4A1*		X	X
*HSPA5*		X	X
*ORM1*		X	X
*PDIA3*		X	X

We report the 33 promising plasma candidate biomarkers for meat tenderness identified in this study. In brackets in the QTL column: chromosome associated with the Tenderness score and/or Shear force QTL. The first 29 promising candidates were selected when located in tenderness QTL (*n* = 24) and/or identified (*n* = 5) in [67]. The four plasma proteins reported at the bottom of table were obtained by overlapping between the BPA and the list of 67 putative muscle biomarkers published in [67]. These four proteins were predicted as secreted proteins (conventional pathways) using ProteINSIDE. “X” means that the protein was found in the Picard and Gagaoua 2019 and/or identify as promising candidate biomarkers.

## Supplementary Materials

Supplementary materials can be found at https://www.mdpi.com/1422-0067/21/2/664/s1; Supplementary Data 1. Reactome representation of the canonical pathways associated with the 13 “predicted secreted proteins in plasma” identified in this study; Supplementary Data 2. Reactome representation of the canonical pathways associated with the 44 “hypothetically secreted proteins in plasma” identified in this study; Supplementary Data 3. List of the 1101 Gene Names used as Bovine Proteome Atlas (BPA).

## Abbreviations

BP	Biological process
BPA	Bovine proteome atlas
EVs	Extracellular vesicles
GN	Gene name
GO	Gene ontology
HPA	Human protein atlas
ID	Identifiers
PPi	Protein-protein interaction
QTL	Quantitative trait loci
UPS	Unconventional pathways of secretion
